# Phylogenetic, Sequencing, and Mutation Analysis of SARS-CoV-2 Omicron (BA.1) and Its Subvariants (BA.1.1, BA.2) During the Fifth Wave of the COVID-19 Pandemic in the Iraqi Kurdistan Region

**DOI:** 10.7759/cureus.48637

**Published:** 2023-11-10

**Authors:** Sherzad M Taher, Jassim M Abdo, Muayad A Merza

**Affiliations:** 1 Department of Basic Sciences, University of Duhok, Duhok, IRQ; 2 Department of Internal Medicine, University of Duhok, Duhok, IRQ

**Keywords:** omicron variants and subvariants, phylogenetic tree, mutations, genome sequence, sars-cov-2, covid-19

## Abstract

Introduction

In December 2019, a global outbreak of SARS-CoV-2 occurred in Wuhan, China, resulting in the COVID-19 pandemic. Since then, the virus has spread to all countries, necessitating a worldwide initiative to create effective treatments and vaccines.

Methods

The RNA of samples QIAamp Viral RNA Mini Kit (Qiagen, MD). SARS-CoV-2 RNA was reverse transcribed with SuperScript IV VILO (ThermoFisher Scientific, Waltham, MA). The virus cDNA was amplified in two multiplexed PCR reactions using Q5 DNA High-fidelity Polymerase (New England Biolabs, Ipswich, MA). The genome was entirely sequenced from 40 samples at the Scripps Research Institute (TSRI) in California, USA. The samples were sequenced using a NovaSeq 6000 SP Reagent Kit v1.5 (Illumina, USA). The TSRI then entered these sequences into the GISAID database. The virus sequence was matched to the SARS-COV-2 virus identified in Wuhan, China (accession number: NC 045512.2) using Illumina sequencing technology (Illumina, CA), finding 95 different changes. The NextClade (clades.nextstrain.org) and Mega 11 (https://www.megasoftware.net) software tools were used to analyze SARS-CoV-2 genome sequence alignment and mutation studies.

Results

Following a sequencing analysis, it was determined that the spike glycoprotein (S) included a total of 38 mutations. Thirty of these mutations were found in the ORF1a gene. Additionally, 11 mutations were found in the ORF1b gene, with the remaining mutations found in the nucleocapsid (N), membrane protein (M), open reading frames 6 (ORF6), open reading frames 9 (ORF9), and envelope (E) genes. The phylogenetic analysis and transmission studies indicated that the isolates discovered in Iraq had separate infection origins and were closely linked to those discovered in other nations and states.

Conclusion

According to the findings of this study, a new vaccine can be developed based on identifying new Omicron variant mutations and subvariants such as BA.2, which were identified for the first time in Iraq.

## Introduction

A global outbreak of SARS-CoV-2 occurred in Wuhan, China, in December 2019, resulting in the COVID-19 pandemic. This sudden epidemic prompted a global effort to develop treatments and vaccines. According to the World Health Organization, as of November 2021, there have been around 260 million confirmed cases of COVID-19, with more than five million deaths worldwide. SARS-CoV-2 is a virus that has a genome of 30 kb and almost 9860 amino acids, with a positive-sense single-stranded RNA structure [1].

The urgency of the epidemic has prompted scientists to dedicate various efforts toward producing effective vaccines. However, viruses, like any other infection, tend to spread more easily in open-air environments. The increase in visitors to crowded areas was less shocking than expected, given the current state of health. Random mutations occur during the replication of SARS-CoV-2 due to the lack of a proofreading mechanism, leading to the formation of numerous strains that have now spread globally [2]. Next-generation sequencing NGS technique is commonly used to sequence the genome of SARS-CoV-2 [3]. The EpiCoV database comprises nearly 12 million whole-genome sequences (WGS) of SARS-CoV-2, which are publicly accessible through the Global Initiative for Sharing All Influenza Data (GISAID). Presently, GISAID recognizes 11 clades based on mutations shared by markers, with L and S emerging early on during the pandemic. The observation of the evolution and transmission of SARS-CoV-2 became possible because of the rapid generation of its genome. The viruses circulating in various regions began to diverge and create separate lineages caused by mutations that accumulated during the viral genome replication and spread among susceptible individuals [4]. Various nomenclature systems are currently used to track the genetic lineages of SARS-CoV-2 at local and global levels. These include WHO labels, GISAIDs, NextStrains, and Pango lineages [5].

The World Health Organization (WHO) has identified four different variants of concern (VOCs). The first variant, B.1.1.7, was initially detected in the United Kingdom. The second variant, P.1, is a descendant of the gamma variant (B.1.1.28) and was first discovered in South Africa in October 2020. The third and fourth variants are the Beta (B.1.351) and the Delta (B.1.617.2), first identified in Brazil and India, respectively [6]. The COVID-19 pandemic affected Iraqi Kurdistan in three waves: the first occurred from March to December 2020, the second from January to June 2021, and the third from July to December 2021 [7]. The Alpha and Beta variants were the most dangerous during the first two waves, while the Delta variant predominated in the third wave [8]. In Iraq, the death rates for the first, second, and third waves were 2.15%, 0.58%, and 0.92%, respectively [9]. Despite the severity of the Delta variant, fatality rates were lower during the third wave [10], which can be related to a lack of experience in managing COVID-19 cases and limited healthcare facilities during the initial stage of the pandemic. In January 2022, five cases of the Omicron variety were recorded in a family in Duhok after a member returned from overseas [11].

It is essential to conduct continuous genomic surveillance to understand better and respond effectively to the potential epidemiological consequences of new mutations. In our study, we analyzed 40 viral genomes of SARS-CoV-2. As a result, we could identify prevalent variations, clades, and lineages and understand the virus better by identifying mutation patterns.

## Materials and methods

Setting

Duhok COVID-19 Hospital is the main tertiary care referral hospital for COVID-19 cases in the Duhok Governorate. It consisted of 50 ward beds and 20 ICU beds. The primary goal of the hospital is to manage severe, critical, and complicated cases.

The University of Duhok (UoD) COVID-19 Center for Research and Diagnosis is an independent center under the University of Duhok, which was officially launched in 2021 following endorsement by the Ministry of Higher Education and Scientific Research and the Ministry of Health of the Kurdistan Region of Iraq.

Clinical sample and processing, sampling, extraction of viral RNA, and performing real-time PCR

A nasopharyngeal swab was collected from 40 COVID-19 patients and was preceded at the COVID-19 Center for Research and Diagnosis, University of Duhok, in December 2021. The chosen samples were extracted and evaluated quantitatively with a virus detection system called QIAprep & Viral RNA UM Kit (Qiagen, MD), which uses RNA extraction and real-time PCR. The cycle threshold (CT) value of the selected samples was below 20, which was required by the sequencing laboratory. The samples were more than 20 which was excluded from the study. The RNA was then extracted from the viral transport medium using the QIAamp Viral RNA Mini Kit (Qiagen, Venlo, The Netherlands).

SARS-CoV-2 RNA (2 µL) was reverse transcribed with SuperScript IV VILO (ThermoFisher Scientific, Waltham, MA). The Scripps Research Institute (TSRI) in La Jolla, California, was in charge of the genome sequencing of the 40 samples.

The virus cDNA was amplified in two multiplexed PCR reactions using Q5 DNA high-fidelity polymerase (New England Biolabs, Ipswich, MA). The libraries were purified with AMPureXP beads and quantified using the Qubit High Sensitivity DNA assay kit (Invitrogen, Waltham, MA) and Tapestation D5000 tape (Agilent, Santa Clara, CA). The samples were sequenced using a NovaSeq 6000 SP Reagent Kit v1.5 (300 cycles) (Illumina, Inc., San Diego, CA).

Whole genome sequencing of the Omicron variant of SARS-CoV-2

As previously stated, the genome was entirely sequenced from 40 samples at the Scripps Research Institute (TSRI) in California, USA. The TSRI then entered these sequences into the GISAID database, where they were assigned accession numbers (see the Appendices Table *3*).

Genome alignment and phylogenetic analysis

SARS-CoV-2 genome sequence alignment and mutation analyses were performed using the NextClade (clades.nextstrain.org) and GISAID database tools to determine lineage and clade assignments, and the pangolins program (version v.3.1.7) was utilized. MEGA 11 (https://www.megasoftware.net) was used to build phylogenetic trees. To construct a phylogenetic tree, we compared the accession numbers of 40 hCoV-19/Iraq/KR sequences to the GISAID database's closest SARS-CoV-2 genome sequences.

## Results

The SARS-COV-2 Omicron variant (BA.1) and its subvariants (BA.1.1, BA.2) were sequenced in Duhok, Iraq, and at the Scripps Research Institute (TSRI) in California from 40 samples. A study sequencing the virus's genome has revealed that the spike glycoprotein (S) has undergone 38 mutations, with 30 of these mutations occurring in the ORF1a gene. Additionally, eleven mutations were found in the ORF1b gene. In contrast, the remaining mutations were located in the nucleocapsid (N), membrane protein (M), Open Reading Frames 6 (ORF6), Open Reading Frames 9 (ORF9), and Envelope (E) genes (Table 1). 

**Table 1:** In this work, we studied 40 SARS-CoV-2 Omicron variant isolates to see how mutations were distributed and how they affected amino acids. Using the Nextclade version tool (version 2.8.0), we aligned the complete genome sequences with the SARS-CoV-2 reference genome (NC045512.2). - = Deletion; * = Substitution

Mutation	Position	Nucleotide change	Code	Amino acid change	Type of mutation		
ORF1a (266...13468)							
	444	GTT>GCT	V60A	Valin>Alanine	Non-synonymous SNV		
	593	CAT>TAT	H110Y	Histidine>Tyrosine	Non-synonymous SNV		
	670	AGT>AGG	S135R	Serine>Arginine	Non-synonymous SNV		
	1415	CTT>TTT	L384F	Leucine>Phenylalanine	Non-synonymous SNV		
	2790	ACT>ATT	T842I	Threonine>Isoleucine	Non-synonymous SNV		
	2832	AAG>AGG	K856R	Lysine>Arginine	Non-synonymous SNV		
	2883	TGT>TAT	C873Y	Cisteine>Tyrosine	Non-synonymous SNV		
	3896	GTT>TTT	V1211F	Valine>Phenylalanine	Non-synonymous SNV		
	4184	GGT>AGT	G1307S	Glycine>Serine	Non-synonymous SNV		
	4893	ACA>ATA	T1543I	Threonin>Isoleucine	Non-synonymous SNV		
	5007	ACG>ATG	T1581M	Threonin>Methionine	Non-synonymous SNV		
	510–518	ATG>-TG	del82/84	del82/84	Non-frame shift deletion		
	519	ATG>-TG	M85V	Methionine>Valine	Non-synonymous SNV		
	6176	GAT>AAT	D1971N	Aspartic acid>Asparagine	Non-synonymous SNV		
	6513-6515		del2083/2083	del2083/2083	Non-synonymous deletion		
	6516	TTA>-TA	L2084I	Leucine>Isoleucine	Non-synonymous SNV		
	7036	TTA>TTT	L2257F	Leucine>Phenylalanine	Non-synonymous SNV		
	7488	ACT>ATT	T2408I	Threonine>Isoleucine	Non-synonymous SNV		
	8393	GCT>ACT	A2710T	Alanine>Threonin	Non-synonymous SNV		
	9344	CTT>TTT	L3027F	Leucine>Phenylalanine	Non-synonymous SNV		
	9474	GCT>GTT	A3070V	Alanine>Valine	Non-synonymous SNV		
	9534	ACT>ATT	T3090I	Threonine>Isoleucine	Non-synonymous SNV		
	9866	CTT>TTT	L32201I	Leucine>Isoleucine	Non-synonymous SNV		
	10029	ACC>ATC	T3255I	Threonin>Isoleucine	Non-synonymous SNV		
	10323	AAG>AGG	K3353R	Lysine>Arginine	Non-synonymous SNV		
	10449	CCC>CAC	P3395H	Proline>Histidine	Non-synonymous SNV		
	11405	GTC>TTC	V3714F	Valine>Phenylalanine	Non-synonymous SNV		
	11285-11293		del3674/3676	del3674/3676	Non-frame shift deletion		
	11537	ATT>GTT	I3758V	Isoleucine>Valine	Non-synonymous SNV		
	12534	ACT>ATT	T409I	Threonine>Isoleucine	Non-synonymous SNV		
ORF1b (13468...21555)							
	13756	ATA>GTA	I97V	Isoleucine>Valine	Non-synonymous SNV		
	14408	CCT>CTT	P314L	Proline>Leucine	Non-synonymous SNV		
	14821	CCA>TCA	P452S	Proline>Serine	Non-synonymous SNV		
	15641	AAT>AGT	N725S	Asparagine>Serine	Non-synonymous SNV		
	15982	GTA>ATA	V839I	Valine>Isoleucine	Non-synonymous SNV		
	16744	GGT>AGT	G1093S	Glycine>Serine	Non-synonymous SNV		
	17410	GGT>TGT	R1315C	Arginine>Cisteine	Non-synonymous SNV		
	18163	ATA>GTA	I1566V	Isoleucine>Valine	Non-synonymous SNV		
	18433	GAT>CAT	D165H	Aspartic acid>Histidine	Non-synonymous SNV		
	19999	GTT>TTT	V2178F	Valine>Phenylalanine	Non-synonymous SNV		
	20003	GAT>GGT	P2179G	Proline>Glycine	Non-synonymous SNV		
S (21563...25384)							
	21765 - 21770	TACATG>- - -	del69/70	del69/70	Non-synonymous deletion		
	21789	ACT>ATT	T76I	Threonine>Isoleucine	Non-synonymous SNV		
	21846	ACT>ATT	T95I	Threonine>Isoleucine	Non-frame shift deletion		
	21987	GGT>GAT	G142D	Glycine>Aspartic acid	Non-synonymous SNV		
	21987 - 21995		del142/144	del142/144	Non-frame shift deletion		
	21996	TAC>-AC	Y145D	Tyrosine>Aspartic acid	Non-synonymous SNV		
	22194 - 22196	AAT>A--	del211/211	del211/211	Non-synonymous deletion		
	22197	TTA>-TA	L212I	Leucine>Isoleucine	Non-synonymous SNV		
	222000	GTG>GGG	V213G	Valine>Glycine	Non-synonymous SNV		
	22578	GCT>GAT	G339D	Glycine>Aspartic acid	Non-synonymous SNV		
	22599	AGA>AAA	R346K	Arginine>Lysine	Non-synonymous SNV		
	22673	T>C	S371L	Serine>Leucine	Non-synonymous SNV		
	22674	C>T	S373P	Serine>Proline	Non-synonymous SNV		
	22686	TCC>TTC	S375F	Serine>Phenylalanine	Non-synonymous SNV		
	22688	ACT>GCT	T376A	Threonine>Isoleucine	Non-synonymous SNV		
	22786	AGA>AGC	R408S	Arginine>Serine	Non-synonymous SNV		
	22813	AAG>AAT	K417N	Lysine>Asparagine	Non-synonymous SNV		
	22882	AAT>AAG	N440K	Asparagine>Lysine	Non-synonymous SNV		
	22898	GGT>AGT	G446S	Glycine>Serine	Non-synonymous SNV		
	23013	GAA>GCA	E484A	Glutamic acid>isoleucine	Non-synonymous SNV		
	22992	AGC>AAC	S477N	Serine>Asparagine	Non-synonymous SNV		
	22995	ACA>AAA	T478K	Threonine>Lysine	Non-synonymous SNV		
	23040	CAA>CGA	Q493R	Glutamine>Arginine	Non-synonymous SNV		
	23048	G>A	G496S	Glycine>Serine	Non-synonymous SNV		
	23055	A>G	Q498R	Glutamine>Arginine	Non-synonymous SNV		
	23063	AAT>TAT	N501Y	Asparagine>Tyrosine	Non-synonymous SNV		
	23075	TAC>CAC	Y505H	Tyrosine>Histidine	Non-synonymous SNV		
	23202	ACA>AAA	T547K	Threonine>Lysine	Non-synonymous SNV		
	23403	GAT>GGT	D614G	Aspartic acid>Glycine	Non-synonymous SNV		
	23525	CAT>TAT	H655Y	Histidine>Tyrosine	Non-synonymous SNV		
	23599	T>G	N679K	Asparagine>Lysine	Non-synonymous SNV		
	23604	CCT>CAT	P681H	Proline>Histidine	Non-synonymous SNV		
	23854	AAC>AAA	N764K	Asparagine>Lysine	Non-synonymous SNV		
	23948	GAT>TAT	D796Y	Aspartic acid>Tyrosine	Non-synonymous SNV		
	24130	ACC>AAA	N856K	Asparagine>Lysine	Non-synonymous SNV		
	24424	CAA>CAT	Q954H	Glutamine>Histidine	Non-synonymous SNV		
	24469	AAT>AAA	N969K	Asparagine>Lysine	Non-synonymous SNV		
	24503	CCT>TTT	L981F	Leucine>Phenylalanine	Non-synonymous SNV		
ORF3a (25393…26220)							
	25471	GAT>TAT	D27Y	Aspartic acid>Tyrosine	Non-synonymous SNV		
	26060	ACT>ATT	T223I	Threonine>Isoleucine	Non-synonymous SNV		
M (26523... 27191)	26530	GAT>GGT	D3G	Aspartic acid>Glycine	Non-synonymous SNV		
	26577	CAA>GAA	Q19E	Glutamine>Glutamic acid	Non-synonymous SNV		
	26709	GCT>ACT	A63T	Alanine>Threonin	Non-synonymous SNV		
ORF6 (27202…27387)	27269	AAA>-AA	K23*	K23*	Non-synonymous SNV		
	27266 - 27268	TTA>- - -	del22/23	del22/23	Non-frame shift deletion		
ORF9b (28284…28577)	28311	CCC>TCC	P10S	Proline>Serine	Non-synonymous SNV		
N (28274…29533)	28881	AGG>AAA	R203K	Arginine>Lysine	Non-synonymous SNV		
	28882	AGG>AAA	R203K	Arginine>Lysine	Non-synonymous SNV		
	28883	GGA>ACG	G204R	Glycine>Arginine	Non-synonymous SNV		
	28311	CCC>CTC	P13L	Proline>Leucine	Non-synonymous SNV		
	28725	CCT>CTT	P151L	Proline>Leucine	Non-synonymous SNV		
	29000	GGC>AGC	G243S	Glycine>Serine	Non-synonymous SNV		
	29005	CAA>CAC	Q244H	Glutamine>Histidine	Non-synonymous SNV		
	29510	AGT>CGT	S413R	Serine>Arginine	Non-synonymous SNV		

**Figure 1:**
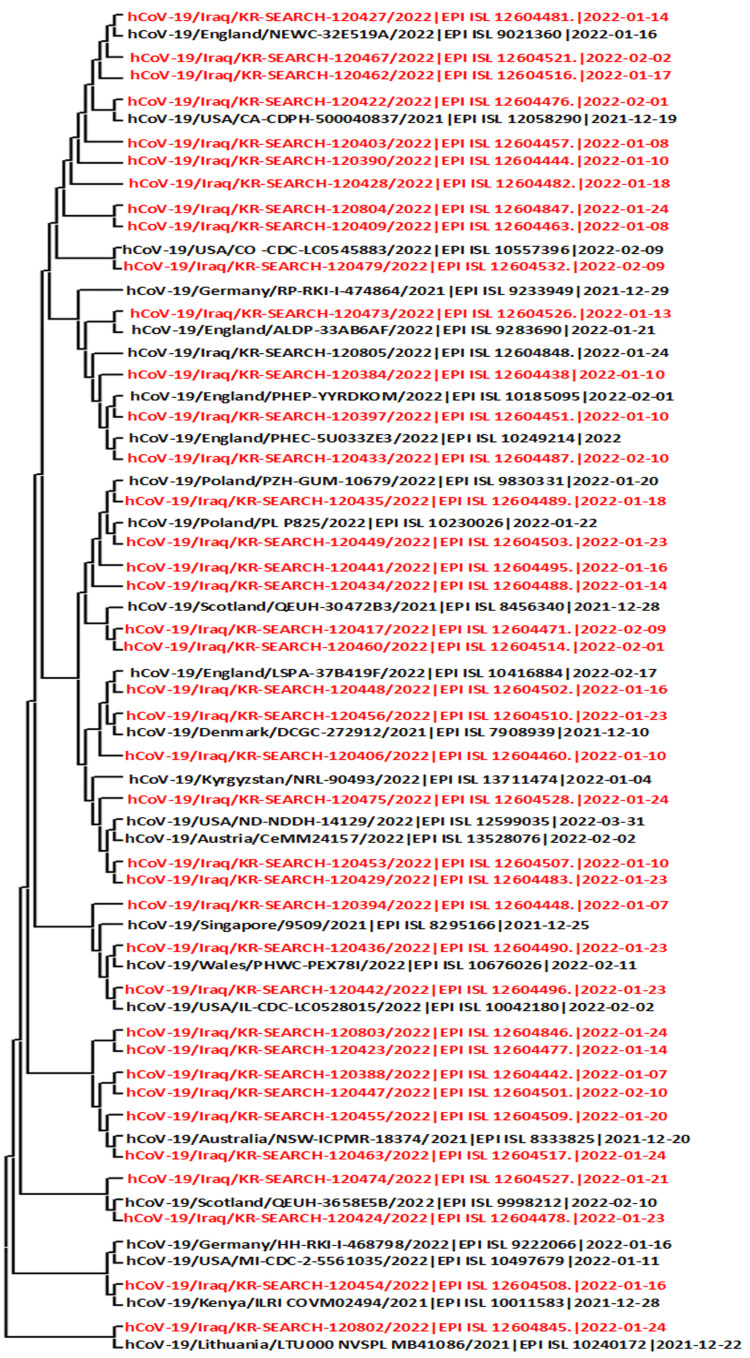
Phylogenetic tree using SARS-COV-2 isolates from various regions and their almost complete gene sequences. The sequences marked in red color were isolated from Duhok province, Iraqi-Kurdistan. The GISAID databases’ websites were used to download the sequence. The MEGA 11 tree-building program employed the neighbor-joining approach.

The phylogenetic analysis and transmission investigations have indicated that the strains isolated in Iraq have distinct infection origins and are closely related to those found in other countries and states (Figure 1).

The genome sequences of the SARS-CoV-2 virus were analyzed and compared with those of several countries, including the United States, United Kingdom, Germany, Austria, Kenya, Poland, Denmark, Kyrgyzstan, Malaysia, Morocco, Singapore, and Lithuania. The results indicate that the isolates studied were closely related to previously sequenced SARS-CoV-2 genomes from Poland, the United States, and the United Kingdom, which belong to the BA.1 lineage. However, isolates from other countries were found to have lineages similar to BA.1.1. Notably, the subvariant BA.2 was found only in Duhok and was isolated from the rest of the world (Table 2).

**Table 2:** The table shows the distribution of closely related genomes to uploaded genomes according to the Pango lineage in the present study. The analyzed data were downloaded from GISAID databases, and the uploaded data in this study were compared with other neighboring countries downloaded from GISAID databases and GenBank. For each query sequence, the table gives the sequence number within the input data and the Accession ID (identified from the name provided in the input data). The other columns provide information on the closest related genome, including the match distance, match quality, accession ID, collections date, submissions date, lineage, and country of origin of the matched genome.

Upload accession ID	Distance	Quality	Accession ID of closest related genome	Lineage	Country/state
EPI_ISL_12604438	1	0.959	EPI_ISL_10185095	BA.1.1	United Kingdom/England
EPI_ISL_12604442	0	0.998	EPI_ISL_12604518	BA.1.1	Iraq/Kurdistan/Duhok
EPI_ISL_12604444	0	0	EPI_ISL_11163451	BA.1.1	USA/Tennessee
EPI-ISL_12604448	0	1	EPI_ISL_11501531	BA.1.1	Canada/Saskatchewan
EPI_ISL_12604478	0	0.974	EPI_ISL_9222066	BA.1.1	Germany/Hamburg
EPI_ISL_12604481	0	0.943	EPI_ISL_9021360	BA.1.1	United Kingdom/England
EPI_ISL_12604482	0	1	EPI_ISL_9041699	BA.1.1	USA/Maryland
EPI_ISL_12604483	0	0.999	EPI_ISL_13528111	BA.1.1	Austria/Styria/Liezen
EPI_ISL_12604487	0	0.977	EPI_ISL_9830331	BA.1	Poland/Swietokrzyskie Voivodeship
EPI_ISL_12604488	0	0.947	EPI_ISL_8456340	BA.1.1	United Kingdom/Scotland
EPI_ISL_12604489	1	1	EPI_ISL_17134891	BA.1	USA/Arkansas
EPI_ISL_12604490	0	0.997	EPI_ISL_10676026	BA.1.1	United Kingdom/Wales
EPI_ISL_12604495	1	0.94	EPI_ISL_11955489	BA.1.1	United Kingdom/England
EPI_ISL_12604496	1	0.942	EPI_ISL_10042180	BA.1.1	USA/Illinois
EPI_ISL_12604501	0	1	EPI_ISL_12604497	BA.1.1	Iraq/Kurdistan/Duhok
EPI_ISL_12604502	0	0.994	EPI_ISL_12604412	BA.1.1	Iraq/Kurdistan/Duhok
EPI_ISL_12604503	0	1	EPI_ISL_11252814	BA.2	USA/Ohio
EPI_ISL_12604507	1	0.999	EPI_ISL_11041477	BA.1.1	United Kingdom/Scotland
EPI_ISL_12604508	0	1	EPI_ISL_10011583	BA.1.1	Kenya/Migori
EPI_ISL_12604509	1	0.954	EPI_ISL_9517295	BA.1.1	Iraq/Baghdad
EPI_ISL_12604510	0	0.991	EPI_ISL_7908939	BA.1.1	Denmark/Syddanmark
EPI_ISL_12604514	1	0.903	EPI_ISL_9835746	BA.1.1	United Kingdom/England
EPI_ISL_12604516	0	1	EPI_ISL_10181069	BA.1.1	United Kingdom/Scotland
EPI_ISL_12604517	0	0.999	EPI_ISL_10497679	BA.1.1	USA/Michigan
EPI_ISL_12604521	0	1	EPI_ISL_17128823	BA.1.1	USA/Arkansas
EPI_ISL_12604527	1	0.986	EPI-ISL_9998212	BA.1.1	United Kingdom/Scotland
EPI_ISL_12604528	1	1	EPI_ISL_13674544	BA.1.1	USA/Minnesota
EPI_ISL_12604532	2	0.989	EPI_ISL_9233949	BA.1.1	Germany/Rhineland-Palatinate
EPI_ISL_12604845	1	0.946	EPI_ISL_10240172	BA.1.1	Lithuania/Kauno apskritis
EPI_ISL-12604846	0	1	EPI_ISL_12604443	BA.1.1	Iraq/Kurdistan/Duhok
EPI_ISL_12604847	0	1	EPI_ISL_9041699	BA.1.1	USA/Maryland
EPI_ISL_12604848	0	1	EPI_ISL_9985416	BA.1.1	Poland/Lodzkie/Lodz
EPI_ISL_12604451	0	0.954	EPI_ISL_10249214	BA.1	United Kingdom/England
EPI_ISL_12604457	1	0.94	EPI_ISL_10557396	BA.1.1	USA/Colorado
EPI_ISL_12604460	2	0.971	EPI_ISL_13711474	BA.1.1	Kyrgyzstan/Chui
EPI_ISL_12604463	1	1	EPI_ISL_12604423	BA.1.1	Iraq/Kurdistan/Duhok
EPI_ISL_12604471	0	0.932	EPI_ISL_9224622	BA.1	Germany/North Rhine-Westphalia
EPI_ISL_12604476 2 0.954	2	0.954	EPI_ISL_12058290	BA.1.1	USA/California/Placer County
EPI_ISL_12604526	0	0.907	EPI_ISL_11405543	BA.1.1	South Korea

**Figure 2:**
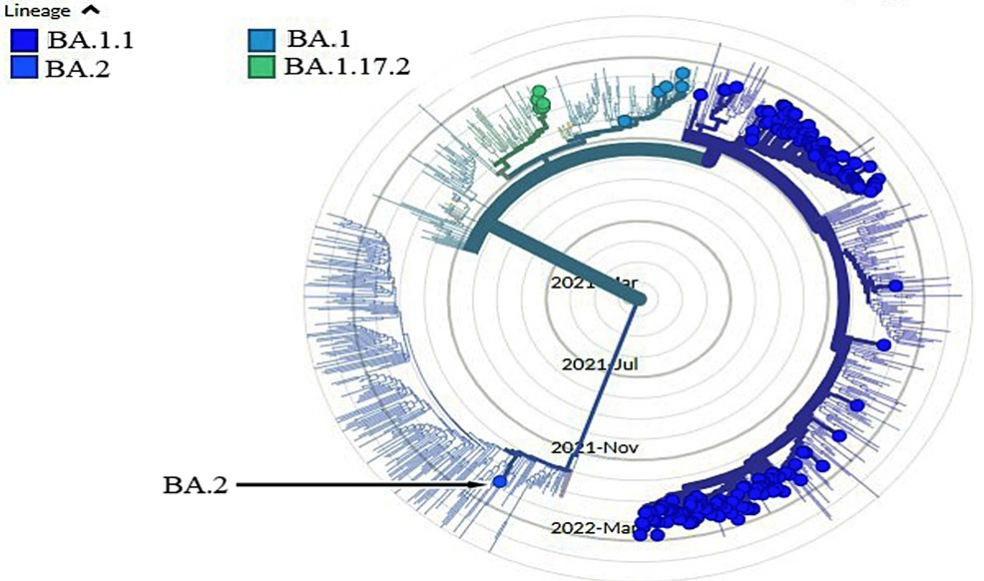
The phylogenetic tree illustrates the distribution of the Pango lineage in Iraq, BA.2 (Accession ID: EPI_ISL_12604503) was first observed in Duhok, Iraq.

The identification of the Omicron BA.2 subvariant in Iraq's fifth wave of COVID-19 cases is a significant and unique discovery (Figure 2).

## Discussion

In late December 2019, multiple cases of pneumonia were reported in China. It was later discovered that a new strain of coronavirus was the cause [12]. SARS-CoV-2 is a virus that has a genome comprising a single-stranded RNA with positive polarity. This means that the orientation of the RNA base sequences is the same as the following messenger RNA (mRNA). SARS-CoV-2 has one of the largest RNA genomes, measuring between 26.4 and 31.7 kilobases. This virus is responsible for causing COVID-19, which has affected millions of people worldwide [13].

**Figure 3:**
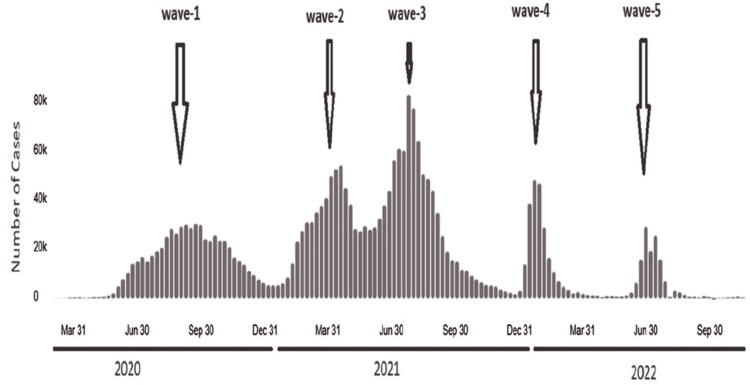
Daily confirmed COVID-19 cases in Iraq from 2021 to 2022, as reported on the WHO dashboard. In Iraq, from January 3, 2020, to October 12, 2023, there have been 2,465,545 confirmed cases of COVID-19 with 25,375 deaths reported to WHO (https://covid19.who.int/region/emro/country/iq) [15].

This article was previously posted at a preprint server (https://www.preprints.org/manuscript/202308.1167) on 14 August 2023 [14]. In April 2022, we uploaded the genome sequences of our isolated viral strain to the GISAID database for analysis between the fourth and fifth waves. The latest COVID-19 wave in Iraq has been linked to the Omicron subvariant (BA.2), according to the coronavirus dashboard of the World Health Organization. This discovery is significant as it has not been previously documented in Iraqi investigations. The finding highlights the potential for SARS-CoV-2 to develop and diversify in Iraq (Figure 3).

Researchers have examined the genetic sequence of the SARS-CoV-2 Omicron variant and found that the S gene has the most mutations, followed by the ORF1ab, N, M, ORF6, ORF3a, ORF9b, and E genes. The genes ORF3a, ORF6, E-gene, and ORF9b have the fewest mutations among these genes. The spike glycoprotein (S) is responsible for the virus's host affinity and pathogenesis; hence, it is the primary target for therapy and diagnostics. The S gene is the structural protein that attaches to the virus and allows host cell receptors to recognize a variety of hosts. Because of the significant mutations found in this gene, it is essential to continuously monitor its evolution to determine its possible impact on public health [16]. This gene contains 38 mutations, with each mutation having the encoded protein's amino acid sequence. T76I, T95I, Y145D, L212I, and V213G are examples of non-synonymous mutations, as are several others whose references are indicated by document numbers. N969K and L981F mutations have also been reported, as have deletions such as del211/211, del3674/3676, del69/70, del142/144, del22/23, del68/70, del142/145, and del211/212. Previous research indicates that this gene has a higher mutation probability than other genomic sites [17]. According to the GISAID SARS-CoV-2 database, as of June 25, 2020, the prevalence of the S protein variant has increased over time. It is now present in 74% of the known variants. A common mutation of the SARS-CoV-2 virus, known as D614G, was initially discovered in China on January 24, 2020. This mutation replaces the polar negatively charged side chain amino acid aspartate (D) with the nonpolar side chain amino acid glycine (G) [18]. D614G significantly enhances transmission, infectivity, and cellular penetration of SARS-CoV-2 in human cells. Positive natural selection drives this [19].

Our study found that some of the most common changes in the RBD improved the binding of ACE2. These changes include N501Y, S477N, and E484K. Additionally, we found that these same substitutions were present in most VOCs, which are associated with greater transmissibility. Furthermore, our study discovered several mutations in T478K, Q498R, and Q493K that increase the electrostatic potential, leading to a stronger binding affinity between RBD and ACE2 [20]. Immune escape has been linked to changes in E484K [21]. A prior investigation found numerous substitutions (E484A) in several VOCs, including B.1.617.2 (E484K/E484Q), B.1.351 (E484K), P.1, and B.1.1.529 [22]. Notably, during the evolution of SARS-CoV-2, the scientists identified numerous mutations, including deletions such as idel69/70 and del142/144. The N-terminal domain (NTD) is a region where deletions frequently occur, including positions 69-70, 141-144, 146, 210, and 243-244. A previous study found that most NTD mutations change the antigenicity or remove epitopes, which may cause immune escape [23].

The mutations of B.1.1.529 have spread extensively, but it is unclear how they affect the severity of the virus and the response of polyclonal mAbs. The ORF1a/b is essential for SARS-CoV-2 nucleic acid tests as the nonstructural proteins produced by ORF1a and ORF1b are necessary for maintaining and replicating the virus [24]. ORF1ab produces two crucial proteins that aid in viral replication: an RNA-dependent RNA polymerase enzyme and a helicase protein. The most frequent changes among the nonstructural proteins in ORF1ab were observed in nsp3, including a deletion at amino acid positions del2083/2083, A 2710 T, and K 856 R. Variants T 3255 I and I 3758 V were found on nsp4 and nsp6, respectively, accompanied with non-frameshift deletions (del3674/3676) [25].

The N-gene, also known as the nucleocapsid protein, is essential for virus detection, antigen and nucleic acid tests, and vaccine production [26]. It is important for the virus's assembly and budding and the host cell's response to viral infection. Its principal role is to maintain the DNA structure within the membrane [27]. The most common forms of N-proteins in the current investigation were found to be the mutations of R203K and G204R, which have been observed in various parts of the world [28]. A previous study has revealed that these mutations in SARS-CoV-2 can lead to increased virulence and transmission. Because of these mutations, the P13L mutation was detected in the current investigation [29].

Mutations in both spike protein and nucleocapsid protein are crucial for the widespread transmission of the virus. The Omicron variation's N gene has multiple deletions that can affect the accuracy of specific diagnostic kits by interfering with primer binding. However, the impact of these changes on the virus's pathogenicity remains unclear. On the other hand, accessory proteins like ORF6 and ORF9b have been discovered to decrease innate immunity, signaling pathways, and interferon (IFN) production by targeting the MAVS adapter linked with mitochondria [30].

Observing the Omicron BA.2 subvariant in Iraq's fifth wave of COVID-19 cases is a remarkable and unique discovery. This discovery highlights the importance of ongoing genomic monitoring initiatives, global data sharing, and reporting of new variants and subvariants. These measures are crucial for motivating prompt public health responses and minimizing the impact of the pandemic.

The SARS-CoV-2 virus has a higher mutation rate than the other strains of concern. This could increase its transmission rate and make it easier for the virus to bypass the immune system, particularly in the spike protein. Due to significant mutations in the immunogenic epitopes of the spike protein, it has become necessary to develop new vaccines incorporating the Omicron strain as a reference. Further research is needed to determine the effectiveness of current vaccines against Omicron and its infectivity rate.

## Conclusions

In this study, 40 SARS-CoV-2 genomes from a clinical sample collected from Duhok, Iraqi Kurdistan, were sequenced. The genome contains additional mutations and known mutations (non-synonymous mutations). Nucleotide sequences contain position-specific mutations such as single-nucleotide variants. Additional analyses were performed on the amino acid substitutions to determine the functional stability of the proteins. Our findings showed that the SARSCoV-2 Omicron variant proteins have 95 different variant locations in their coding regions. The spike protein has the most mutations, followed by six structural proteins (ORF1a, N, M, ORF6, ORF9, and E). We found fewer mutations in ORF6, ORF9, and E proteins than in S proteins and ORF1a. A non-synonymous substitution was observed in all protein variants in the whole-genome sequence. Additionally, we observed an omicron subvariant (BA.2) in Duhok, which has not been previously recorded in other studies from Iraq. The new subvariant (BA.2) findings suggest that public health authorities should strengthen SARS-CoV-2 control strategies by examining mutation patterns in circulating variants. Considering everything, we urge that future studies focus on the impacts and roles of these genetic polymorphisms on virus transmission, disease progression, and intensity. Furthermore, creating novel vaccines, particularly ones containing several variants of concern (VOCs), could aid in the control of recent outbreaks.

## Appendices

**Table 3:** Metadata including sequencing statistics, GISAID accession numbers, and PANGO lineage assignments for the present study samples.

SEARCH sample ID	Original sample ID	Gisaid_accession	location	Host	lineage	lineage_name
SEARCH-120384	631	EPI_ISL_12604438	North America/Iraq/Kurdistan/Duhok	Human	BA.1.1	Omicron-like
SEARCH-120388	524	EPI_ISL_12604442	North America/Iraq/Kurdistan/Duhok	Human	BA.1.1	Omicron-like
SEARCH-120390	632	EPI_ISL_12604444	North America/Iraq/Kurdistan/Duhok	Human	BA.1.1	Omicron-like
SEARCH-120394	525	EPI_ISL_12604448	North America/Iraq/Kurdistan/Duhok	Human	BA.1.1	Omicron-like
SEARCH-120397	633	EPI_ISL_12604451	North America/Iraq/Kurdistan/Duhok	Human	BA.1.1	Omicron-like
SEARCH-120403	527	EPI_ISL_12604457	North America/Iraq/Kurdistan/Duhok	Human	BA.1.1	Omicron-like
SEARCH-120406	635	EPI_ISL_12604460	North America/Iraq/Kurdistan/Duhok	Human	BA.1.1	Omicron-like
SEARCH-120409	528	EPI_ISL_12604463	North America/Iraq/Kurdistan/Duhok	Human	BA.1.1	Omicron-like
SEARCH-120417	625	EPI_ISL_12604471	North America/Iraq/Kurdistan/Duhok	Human	BA.1.1	Omicron-like
SEARCH-120422	626	EPI_ISL_12604476	North America/Iraq/Kurdistan/Duhok	Human	BA.1.1	Omicron-like
SEARCH-120423	644	EPI_ISL_12604477	North America/Iraq/Kurdistan/Duhok	Human	BA.1.1	Omicron-like
SEARCH-120424	668	EPI_ISL_12604478	North America/Iraq/Kurdistan/Duhok	Human	BA.1.1	Omicron-like
SEARCH-120427	645	EPI_ISL_12604481	North America/Iraq/Kurdistan/Duhok	Human	BA.1.1	Omicron-like
SEARCH-120428	657	EPI_ISL_12604482	North America/Iraq/Kurdistan/Duhok	Human	BA.1.1	Omicron-like
SEARCH-120429	669	EPI_ISL_12604483	North America/Iraq/Kurdistan/Duhok	Human	BA.1.1	Omicron-like
SEARCH-120433	628	EPI_ISL_12604487	North America/Iraq/Kurdistan/Duhok	Human	BA.1	Omicron-like
SEARCH-120434	646	EPI_ISL_12604488	North America/Iraq/Kurdistan/Duhok	Human	BA.1.1	Omicron-like
SEARCH-120435	658	EPI_ISL_12604489	North America/Iraq/Kurdistan/Duhok	Human	BA.1	Omicron-like
SEARCH-120436	670	EPI_ISL_12604490	North America/Iraq/Kurdistan/Duhok	Human	BA.1.1	Omicron-like
SEARCH-120441	647	EPI_ISL_12604495	North America/Iraq/Kurdistan/Duhok	Human	BA.1.1	Omicron-like
SEARCH-120442	671	EPI_ISL_12604496	North America/Iraq/Kurdistan/Duhok	Human	BA.1.1	Omicron-like
SEARCH-120447	630	EPI_ISL_12604501	North America/Iraq/Kurdistan/Duhok	Human	BA.1.1	Omicron-like
SEARCH-120448	648	EPI_ISL_12604502	North America/Iraq/Kurdistan/Duhok	Human	BA.1.1.4	Omicron-like
SEARCH-120449	672	EPI_ISL_12604503	North America/Iraq/Kurdistan/Duhok	Human	BA.2	Omicron-like
SEARCH-120453	636	EPI_ISL_12604507	North America/Iraq/Kurdistan/Duhok	Human	BA.1.1	Omicron-like
SEARCH-120454	649	EPI_ISL_12604508	North America/Iraq/Kurdistan/Duhok	Human	BA.1.1	Omicron-like
SEARCH-120455	661	EPI_ISL_12604509	North America/Iraq/Kurdistan/Duhok	Human	BA.1.1	Omicron-like
SEARCH-120456	673	EPI_ISL_12604510	North America/Iraq/Kurdistan/Duhok	Human	BA.1.1	Omicron-like
SEARCH-120460	621	EPI_ISL_12604514	North America/Iraq/Kurdistan/Duhok	Human	BA.1.1	Omicron-like
SEARCH-120462	651	EPI_ISL_12604516	North America/Iraq/Kurdistan/Duhok	Human	BA.1.1	Omicron-like
SEARCH-120463	675	EPI_ISL_12604517	North America/Iraq/Kurdistan/Duhok	Human	BA.1.1	Omicron-like
SEARCH-120467	622	EPI_ISL_12604521	North America/Iraq/Kurdistan/Duhok	Human	BA.1.1	Omicron-like
SEARCH-120473	641	EPI_ISL_12604526	North America/Iraq/Kurdistan/Duhok	Human	BA.1.1	Omicron-like
SEARCH-120474	665	EPI_ISL_12604527	North America/Iraq/Kurdistan/Duhok	Human	BA.1.1	Omicron-like
SEARCH-120475	677	EPI_ISL_12604528	North America/Iraq/Kurdistan/Duhok	Human	BA.1.1	Omicron-like
SEARCH-120479	624	EPI_ISL_12604532	North America/Iraq/Kurdistan/Duhok	Human	BA.1.1	Omicron-like
SEARCH-120802	678	EPI_ISL_12604845	North America/Iraq/Kurdistan/Duhok	Human	BA.1.1	Omicron-like
SEARCH-120803	679	EPI_ISL_12604846	North America/Iraq/Kurdistan/Duhok	Human	BA.1.1	Omicron-like
SEARCH-120804	680	EPI_ISL_12604847	North America/Iraq/Kurdistan/Duhok	Human	BA.1.1	Omicron-like
SEARCH-120805	681	EPI_ISL_12604848	North America/Iraq/Kurdistan/Duhok	Human	BA.1.1	Omicron-like
